# Tick-borne pathogens induce differential expression of genes promoting cell survival and host resistance in *Ixodes ricinus* cells

**DOI:** 10.1186/s13071-017-2011-1

**Published:** 2017-02-15

**Authors:** Karen L. Mansfield, Charlotte Cook, Richard J. Ellis, Lesley Bell-Sakyi, Nicholas Johnson, Pilar Alberdi, José de la Fuente, Anthony R. Fooks

**Affiliations:** 1 Animal and Plant Health Agency (APHA), Woodham Lane, New Haw, Surrey KT15 3NB UK; 2 Institute of Infection and Global Health, University of Liverpool, Liverpool, UK; 3 The Tick Cell Biobank, The Pirbright Institute, Ash Road, Pirbright, Woking, Surrey GU24 0NF UK; 4 Faculty of Health and Medicine, University of Surrey, Guildford, Surrey GU2 7XH UK; 5 SaBio, Instituto de Investigación en Recursos Cinegéticos IREC-CSIC-UCLM-JCCM, Ronda de Toledo s/n, Ciudad Real, 13005 Spain; 6 Department of Veterinary Pathobiology, Center for Veterinary Health Sciences, Oklahoma State University, Stillwater, OK 74078 USA; 7 Department of Clinical Infection, Microbiology and Immunology, University of Liverpool, Liverpool, UK

**Keywords:** Tick cell, Flavivirus, *Anaplasma phagocytophilum*, *Ixodes ricinus*, Apoptosis, Toll, Transcriptomics, Immunology

## Abstract

**Background:**

There has been an emergence and expansion of tick-borne diseases in Europe, Asia and North America in recent years, including Lyme disease, tick-borne encephalitis and human anaplasmosis. The primary vectors implicated are hard ticks of the genus *Ixodes*. Although much is known about the host response to these bacterial and viral pathogens, there is limited knowledge of the cellular responses to infection within the tick vector. The bacterium *Anaplasma phagocytophilum* is able to bypass apoptotic processes in ticks, enabling infection to proceed. However, the tick cellular responses to infection with the flaviviruses tick-borne encephalitis virus (TBEV) and louping ill virus (LIV), which cause tick-borne encephalitis and louping ill respectively, are less clear.

**Results:**

Infection and transcriptional analysis of the *Ixodes ricinus* tick cell line IRE/CTVM20 with the viruses LIV and TBEV, and the bacterium *A. phagocytophilum,* identified activation of common and distinct cellular pathways. In particular, commonly-upregulated genes included those that modulate apoptotic pathways, putative anti-pathogen genes, and genes that influence the tick innate immune response, including selective activation of toll genes.

**Conclusion:**

These data provide an insight into potential key genes involved in the tick cellular response to viral or bacterial infection, which may promote cell survival and host resistance.

**Electronic supplementary material:**

The online version of this article (doi:10.1186/s13071-017-2011-1) contains supplementary material, which is available to authorized users.

## Background

The past few decades have seen the emergence and expansion of tick-borne diseases in Europe, Asia and North America [1]. These include Lyme disease, tick-borne encephalitis, human granulocytic anaplasmosis (HGA) and louping ill [2–5]. Hard ticks of the genus *Ixodes* are primarily implicated, including *Ixodes ricinus*, *Ixodes persulcatus* and *Ixodes scapularis*.

The flavivirus tick-borne encephalitis virus (TBEV) is responsible for an increasing number of cases of human tick-borne encephalitis (TBE) throughout Europe and Asia. Infection with the virus can cause severe encephalitic disease which can be fatal in some cases [6]. Louping ill virus (LIV), which is genetically and antigenically closely-related to TBEV, also causes severe encephalitis and is responsible for disease in sheep in the United Kingdom (UK) and discrete parts of mainland Europe [5]. Both viruses are transmitted by *I. ricinus,* although in Asia, TBEV is transmitted predominantly by *I. persulcatus* [7]. *Anaplasma phagocytophilum* is considered an emerging zoonotic bacterium, transmitted by *I. ricinus* ticks in Europe, and *I. scapularis* in the United States [8]. *Anaplasma phagocytophilum* infects vertebrate host granulocytes, leading to human, canine or equine granulocytic anaplasmosis and to tick-borne fever in ruminants [9–11].

The biological effect on *I. ricinus* ticks of infection with these pathogens has yet to be fully characterised, and genes associated with apoptosis and innate immune function are of particular interest, as these pathways are crucially involved in the cellular response to infection. The induction of apoptosis serves a range of functions in the vertebrate host, including control at the cellular level following infection [12]. Previous studies have shown that *A. phagocytophilum* is able to inhibit this process in *I. scapularis* ticks and human cells, through inhibition of different apoptotic pathways, leading to increased bacterial dissemination [13]. Subsequent studies have shown that the transcriptional response to *A. phagocytophilum* infection in an *I. ricinus* cell line was similar to that detected in *I. scapularis* midguts [14, 15], where the response did not associate the intrinsic apoptotic pathway with the inhibition of cellular apoptosis, but did suggest a role for the janus-associated kinase-signal transducer and activator of transcription (Jak-STAT) pathway *via* upregulation of Jak [15]. Along with the Jak-STAT pathway, the Toll pathway is known to constitute part of the innate immune response in arthropods [16]. A number of recent studies have investigated the response of tick cells to virus infection and provided preliminary data on the pathways activated by flaviviruses [17–19].

In this study, the transcriptional response of an *I. ricinus* cell line to LIV and TBEV infection was investigated, and compared to that observed following *A. phagocytophilum* infection. All infection experiments were undertaken simultaneously, and the dataset derived from *A. phagocytophilum* infection has previously been utilised to investigate apoptosis in a comparison with infection in *I. scapularis* cells [15]. The utilisation of a systems biology approach using high-throughput omics technology has enabled the generation of large datasets yielding evidence of differential gene expression associated with both apoptotic and innate immune pathways. Furthermore, evidence for increased expression of anti-pathogen genes is demonstrated. The application of Next Generation Sequencing (NGS) and subsequent transcriptomic analysis has provided an insight into the tick cell response to virus or bacterial infection, and enhanced our understanding of the tick-pathogen interface.

## Methods

### Virus and bacterial isolates

The virus isolates used were LIV strain LI3/1 (APHA reference: Arb 126), which was originally isolated from a sheep in Oban, Scotland, in 1962, and the TBEV strain Neudorfl H2J (APHA reference: Arb 131), originally isolated from an *I. ricinus* tick in Austria in the early 1950s. Both isolates were mouse brain homogenates, kindly provided by Professor John Stephenson (Public Health England, formerly Centre for Applied Microbiology and Research, Porton Down, UK). The TBEV isolate was originally isolated by Dr Christian Kunz, University of Vienna, Austria, and had subsequently been passaged four times in an outbred strain of mice. However, it remains genetically identical to the standard prototype Neudoerfl strain. The LIV isolate was originally isolated by Dr Hugh Reid, Moredun Institute, Scotland, and had been passaged four times in sheep and six times in an outbred strain of mice. The bacterial isolate was *A. phagocytophilum* NY-18, which was originally isolated from a human in 1996 [20, 21]. The isolate was subsequently passaged in *I. scapularis* tick cells prior to infection of *I. ricinus* cells.

### *Ixodes ricinus* cell line

The *I. ricinus* embryo-derived tick cell line IRE/CTVM20 [22] (provided by the Tick Cell Biobank, The Pirbright Institute, UK) was maintained in a 1:1 mixture of supplemented L-15 (Leibovitz) medium and L-15B medium [23], as previously described [24]. Briefly, the supplemented L-15 medium contained 20% foetal bovine serum (FBS), 10% tryptose phosphate broth (TPB), 2 mM L-glutamine, 100 μg/ml streptomycin and 100 U/ml penicillin. The L-15B medium included 10% TPB, 5% FBS, 0.1% bovine lipoprotein concentrate, 2 mM L-glutamine, 100 μg/ml streptomycin, 100 U/ml penicillin and a range of vitamin and mineral supplements, as previously described [23].

### Infection of tick cells with bacterial and viral isolates

IRE/CTVM20 cells were seeded in 12-well tissue culture plates (Greiner), at a cell density of 6 × 10^5^ cells/well. Quadruplicate wells were infected with either louping ill virus (LIV), tick-borne encephalitis virus (TBEV) or *A. phagocytophilum* at a multiplicity of infection (MOI) of 1, alongside uninfected control wells. Plates were incubated at 28 °C without additional CO_2_; this was the optimal temperature for growth of this cell line, as utilised in previous studies [24–26]. Samples were taken at 0, 72, 120 and 168 hours post-infection (hpi), and cells and medium within each well were resuspended and transferred to sterile cryotubes at each time-point. Following centrifugation at 2000 rpm (366× *g*) for 15 min, the supernatants were transferred to fresh tubes for virus titration. The cell pellets were mixed with 0.5 ml of TRIzol® reagent (Invitrogen, Life Technologies, Paisley, UK) for RNA extraction.

### Extraction of RNA from infected tick cells

Total RNA was extracted from the cell pellets combined with TRIzol® reagent (Invitrogen), according to the manufacturer’s instructions. The RNA was further purified using an RNeasy Mini kit (QIAgen, Manchester, UK), according to the manufacturer’s instructions, and quantified spectrophotometrically. Each RNA sample was assessed by RT-PCR for evidence of virus replication prior to further treatment of the remaining RNA in preparation for sequencing.

### Confirmation of virus growth in tick cells

To assess the growth of LIV and TBEV in tick cells, both the cells and the cell supernatants were assessed at each time-point. To assess growth in cells, extracted RNA was reverse-transcribed using Moloney Murine Leukemia Virus Reverse Transcriptase (M-MLV RT; Promega, Southampton, UK) and random hexamers (Roche, Burgess Hill, UK), according to the manufacturers’ instructions. Positive controls were included, using RNA previously extracted from the virus isolates used as inoculum. Molecular standards with known copy numbers were prepared from the positive control cDNA samples, and were used to quantify the viral copy numbers in the test samples by qPCR using SYBR Green Jumpstart Taq Readymix (Sigma-Aldrich, Gillingham, UK) and TBEV/LIV-specific primers. Similarly, host standards were prepared from RNA extracted from uninfected IRE/CTVM20 cells. Acari primers (Additional file 1: Table S1) were used to quantify 16S copies in the test samples, to allow normalisation of results. To quantify virus titre of the cell supernatants at each time-point, the supernatants were assessed by plaque assay on Vero C1008 cells, using standard techniques [27].

### Confirmation of bacterial growth in tick cells

Growth of *A. phagocytophilum* in tick cells was confirmed by qPCR and microscopy of Giemsa-stained slides. DNA was extracted from tick cells using Tri Reagent (Sigma-Aldrich) following the manufacturer’s instructions*. Anaplasma phagocytophilum* DNA levels were characterised by major surface protein 4 gene (*msp4*) real-time PCR with normalisation against the level of tick 16S ribosomal RNA (rRNA) as described previously [13]. Giemsa-stained cytocentrifuge smears were prepared from resuspended cell suspensions, and were inspected microscopically to monitor infection.

### Next generation sequencing (NGS)

rRNA was depleted from extracted total RNA, using Terminator exonuclease (Epicentre [Illumina], Madison, USA) according to the manufacturer’s instructions. Prior to sequencing, the RNA was quantified (Nanodrop, Nanodrop Products, Thermo Scientific), to confirm that it was at a suitable concentration for Illumina sequencing (> 10 ng/μl).

Sequencing was undertaken of duplicate RNA samples from 0 hpi uninfected and 168 hpi infected cells only. RNA (200 ng) was reverse-transcribed to generate double-stranded cDNA, using the cDNA Synthesis System (Roche) and random hexamers. Illumina sequencing libraries were prepared using the Nextera XT system (Illumina, Madison, USA), and these were sequenced using an Illumina GA IIx instrument.

### Bioinformatics and determination of differential gene expression

Sequence analysis was undertaken using multiplexed paired-end samples. Pre-analysis sequence quality checking was performed using the ‘FastQC’ programme (Barham Institute). The *Ixodes scapularis* genome was used as a reference for mapping (Broad Institute assembly lscaW1), and the programme ‘BowTie 2’ was used as an assembler, to align sequenced reads with the reference sequence. ‘TopHat’ was used to analyse the mapping results and identify splice junctions between exons. The ‘Cufflinks’ programme was used to provide an estimation of gene and isoform abundance and differential expression, allowing for splice variants and gaps due to the genome reference. Within Cufflinks, ‘Cuffmerge’ was used to merge Cufflinks assemblies, to provide normalisation of biological replicates. ‘Cuffquant’ was used to provide abundance estimation across normalised samples. The ‘Cuffdiff’ algorithm was used to account for biological variability between samples and identify differentially expressed genes; this included non-statistical analysis (log fold-change) and statistical analysis (test for variance), in order to identify statistically significant fold-changes in gene expression (*P* < 0.05, *q* < 0.05).

Genome coverage and depth statistics were generated using the ‘Qualimap’ programme [28, 29].

RNAseq data derived from LIV and TBEV-infected tick cells have been deposited in NCBI’s Gene Expression Omnibus, and are available through GEO Series accession number (http://www.ncbi.nlm.nih.gov/geo/query/acc.cgi?acc=GSE85300). RNAseq data derived from *A. phagocytophilum*-infected tick cells has been previously analysed and published [15], and is available through GEO Series accession number (http://www.ncbi.nlm.nih.gov/geo/query/acc.cgi?acc=GSE76906).

### Molecular detection of transcripts

Selected differentially-expressed genes were detected by qPCR using specifically-designed primer pairs, and the primer sequences are detailed in Additional file 1: Table S1. The genes selected were Toll genes (ISCW022740, ISCW007727, ISCW007724 and ISCW017724) and Myeloid differentiation factor 88 (*MyD88*) gene (ISCW008802), along with *Acari* 16S rRNA as a housekeeping gene. Briefly, RNA was reverse transcribed with M-MLV RT (Promega) and random hexamers (Roche), in the presence of 14 units of RNAsin and 10 mM dithiothreitol (DTT), with incubation for 60 min at 42 °C.

Amplification using transcript-specific primer sets was performed using SYBR® Green JumpStart^TM^ Taq Readymix^TM^ (Sigma-Aldrich) and an Mx3005p (Stratagene [Agilent], Santa Clara, USA). Amplification was achieved using specific primers for each mRNA transcript, which was quantified by comparison with a standard curve. The amount of 16S rRNA transcript was used to normalise each transcript within the sample. Transcript fold changes were calculated relative to the uninfected control cells at 0 hpi as previously described [30], with three test groups, *A. phagocytophilum*, LIV and TBEV, sampled at 72 hpi and 120 hpi. Results are reflected as transcript mean fold-change, with standard error of the mean (SEM). Upregulated transcripts have fold-change > 1, and downregulated transcripts have fold-change < 1. Statistical analysis was performed using a Student’s *t*-test with unequal variance. Statistically significant differences between the results from infected samples in comparison to the uninfected controls were denoted as follows: *P < 0.05; **P < 0.01.

## Results

### Pathogen growth in tick cells

**Fig. 1:**
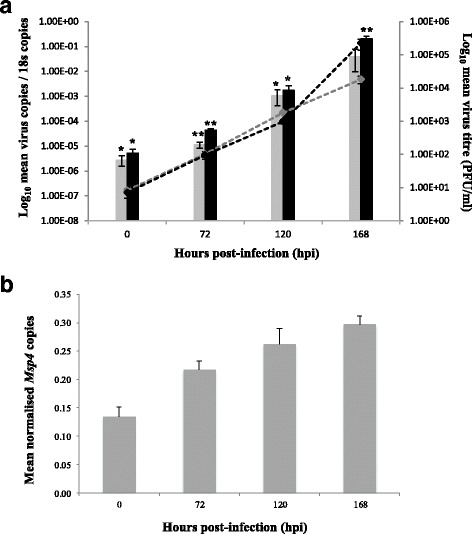
**a** Replication of LIV (*grey*) and TBEV (*black*) in IRE/CTVM20 cells, as demonstrated by log_10_ mean virus copies/16S copies detected in cellular-derived RNA (dashed lines), and virus titre (PFU/ml) quantified in cell supernatant (solid bars). **b** Replication of *Anaplasma phagocytophilum* in IRE/CTVM20 cells, as demonstrated by mean *Msp4* copies normalised against mean 16S copies in cellular-derived RNA. Error bars represent the standard deviation. *P < 0.05; **P < 0.01

Infection of IRE/CTVM20 cells with LIV, TBEV or *A. phagocytophilum* did not appear to inhibit cell growth or alter the cell morphology over the course of the experiment, as previously demonstrated [18, 25]. These observations were based on comparison between infected and uninfected cells using cell counts and examination of cell morphology under the microscope. Growth of LIV and TBEV was confirmed in both cell supernatants and cells (Fig. 1a) and of *A. phagocytophilum* in cells (Fig. 1b).

### Infection with LIV, TBEV and *A. phagocytophilum* induces differential gene expression in a tick cell line

Initial gene expression analysis using RNA derived from *A. phagocytophilum*-infected tick cells has recently been published [15], but the extensive dataset generated has been further utilised here for comparison with flavivirus-induced gene expression. Infection of IRE/CTVM20 cells with LIV or TBEV induced differential gene expression (*P* < 0.05 and *q* < 0.05) as shown previously for *A. phagocytophilum* infection in the same cell line [15] and TBEV in another *I. ricinus* cell line IRE/CTVM19 [18]. These analyses have identified which of the annotated genes were differentially expressed in response to infection with LIV (samples 17 and 18) and TBEV (samples 19 and 20) when compared to uninfected control cells (samples 1–4). Details of the sequence depth and quality of alignment for these samples are provided in Additional file 2: Table S2, and suggest that the sequencing coverage was generally uniform across all of the samples included in the study, with between 38.34 and 50.65% of aligned pairs being within exon annotated sections of the reference genome. However, as we were not attempting to identify novel transcripts there was no requirement for ultra-deep sequencing. Additionally, the statistics for *A. phagocytophilum* (samples 15 and 16), of which some transcriptomic data has been previously published [15], have also been included in this table for comparison.

**Fig. 2:**
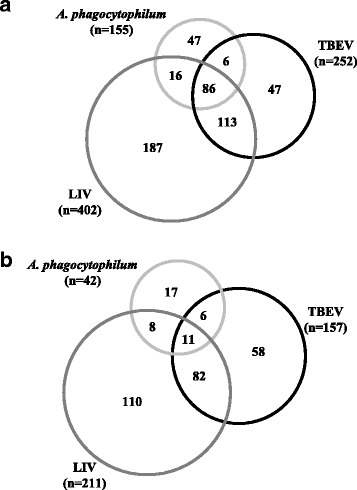
Distribution of differentially upregulated (**a**) and downregulated (**b**) genes, following infection of IRE/CTVM20 cells with *A. phagocytophilum*, LIV or TBEV

**Fig. 3:**
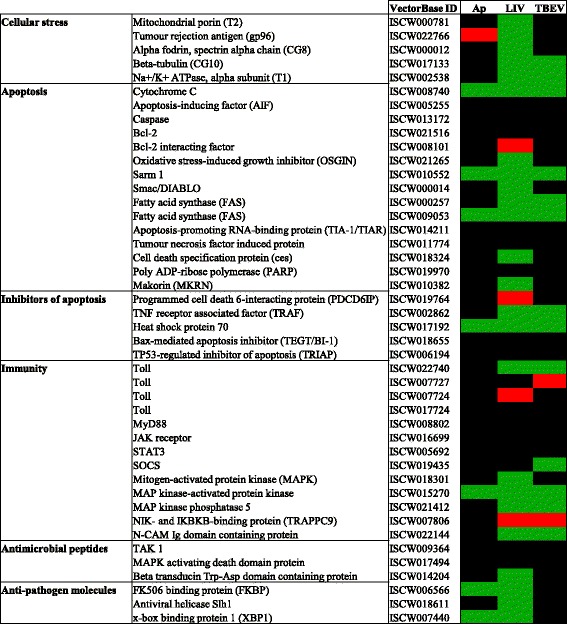
Summary of differential gene expression for selected genes, highlighted *green* (upregulated), *red* (downregulated) or *black* (no significant change), following infection of IRE/CTVM20 cells with *A. phagocytophilum* (Ap), LIV or TBEV

Following LIV and TBEV infection, RNAseq analysis identified 20,861 genes in IRE/CTVM20 cells, which was identical to results reported previously for *A. phagocytophilum* [15] (Table 1). When comparing infected cells at 168 hpi with uninfected cells at 0 hpi, analysis of differential expression identified 613 and 409 differentially expressed genes for LIV and TBEV infection, respectively, in comparison to 197 genes following *A. phagocytophilum* infection, as previously reported [15]. When compared to *A. phagocytophilum* infection, this suggests that flavivirus infection in tick cells leads to the differential expression of a greater number of genes, with 0.95, 2.96 and 1.98% of genes differentially expressed for *A. phagocytophilum*, LIV and TBEV, respectively. Although this suggests differences between the transcriptional responses to each pathogen, the limited number of samples in the study did not enable any correlation between levels of infection and levels of RNA expression to be inferred. However, it is possible that the low infection rate of *A. phagocytophilum* in IRE/CTVM20 cells observed (Fig. 1) may provide an alternative explanation for this difference. Following infection with all three pathogens, the majority of differentially expressed genes in tick cells were upregulated, rather than downregulated, with 78.7, 65.6 and 61.6% of genes upregulated following *A. phagocytophilum*, LIV and TBEV infection, respectively. The numbers of genes shared between pathogens which were significantly upregulated or downregulated are shown in Fig. 2a and b, respectively. Although the vast majority of differentially-expressed genes were involved in metabolic processes, many are important in a number of key pathways, including apoptosis, innate immune response and anti-pathogen responses. Selected key genes involved in these pathways are detailed in Fig. 3 and, again, a key observation is the greater number of genes differentially expressed following flavivirus infection, when compared to *A. phagocytophilum* infection, suggesting that virus infection may have a more profound effect on tick cells than *A. phagocytophilum* infection.

**Table 1:** RNAseq statistics for *I. ricinus* IRE/CTVM20 cells, uninfected at 0 hpi, or infected with LIV or TBEV at 168 hpi (upper panel), and differential gene expression statistics at 168 hpi following infection with LIV or TBEV at 0 hpi, compared to uninfected cells at 0 hpi (lower panel)

Parameter	Uninfected 0 hpi (replicate 1/replicate 2)	LIV 168 hpi (replicate 1/replicate 2)	TBEV 168 hpi (replicate 1/replicate 2)
Total reads	11,976,631/10,334,781	16,075,150/13,693,034	13,339,640/18,910,604
Aligned reads	11,976,631/10,334,781	16,075,150/13,693,034	13,339,640/18,910,604
PF_reads	11,976,631/10,334,781	16,075,150/13,693,034	13,339,640/18,910,604
PF_aligned reads	11,976,631/10,334,781	16,075,150/13,693,034	13,339,640/18,910,604
PF_HQ_aligned reads	3,878,432/3,760,670	4,956,000/3,983,347	4,775,958/6,552,021
Mean read length	119/119	119/119	119/119
PF_HQ_error rate	0.76256/0.76193	0.763221/0.764392	0.766099/0.765034
PF_indel rate	0.000667/0.000668	0.0007/0.000667	0.000629/0.000636
Total genes annotated	na	20,861	20,861
Total DE genes	na	613 (2.96%)	409 (1.98%)
Upregulated DE genes	na	402 (65.6%)	252 (61.6%)
Downregulated DE genes	na	211 (34.4%)	157 (38.4%)

*Abbreviations: na* not available, *HPI* hours post-infection, *PF* pass-Illumina filter, *HQ* high quality, *PF_HQ_ERROR_RATE* percentage of *I. ricinus* sequence bases that mismatch the *I. scapularis* reference genome sequence in PF HQ aligned reads, *PF_INDEL_RATE* number of insertion and deletion events per 100 PF aligned bases. This uses the number of events as the numerator, not the number of inserted or deleted bases. Picard metrics definitions (https://broadinstitute.github.io/picard/picard-metric-definitions.html) were used, *DE* differentially expressed

### Upregulation of key regulators of apoptosis following infection with *A. phagocytophilum*, LIV or TBEV

By 168 hpi for all three pathogens, genes associated with cellular stress and apoptosis were shown to be differentially expressed (*P* < 0.05 and *q* < 0.05) when compared to uninfected cells at 0 hpi (Fig. 3). Significant upregulation of the gene encoding cytochrome *c* following infection with all three pathogens was observed. Crucially however, there was no detection of *Caspase* gene upregulation (Fig. 3) following infection with any of the pathogens. This suggests that not all components of the apoptotic pathway were transcriptionally induced, although this observation may be due to lack of sequence coverage for some genes. Significant upregulation of the gene encoding heat shock protein 70 (Hsp70) was induced by all three pathogens, and may suggest potential involvement in inhibition of apoptosis (Fig. 3) [31].

### Host cell survival supported by expression of innate immune activation and anti-pathogen genes

Viral infection is known to activate the Toll and Jak-STAT pathways [32]. There was evidence for activation of an immune response in *I. ricinus* tick cells *via* the Jak-STAT pathway following infection with all three pathogens, with a number of innate immunity genes differentially expressed at 168 hpi (Fig. 3, Table 2). Only *A. phagocytophilum* infection led to an increase in expression of the Jak receptor gene as previously described [15], although this was not significantly differentially expressed (*P* < 0.05, but *q* > 0.05) (Table 2), and there was also no observed differential expression of the gene encoding STAT3. However, the Jak-STAT modulatory suppressor of cytokine signalling (*Socs*) gene was upregulated significantly following TBEV infection, with some evidence for upregulation following LIV infection (*P* = 0.009, *q* = 0.055). There was also evidence for activation of the mitogen-activated protein kinase (MAPK) pathway; although only LIV infection caused upregulation of the gene encoding MAPK, all three pathogens induced significant upregulation of genes for MAPK-activated protein kinase and MAPK phosphatase 5 (Fig. 3, Table 2).

**Table 2:** RNAseq results for *I. ricinus* IRE/CTVM20 cells infected with *A. phagocytophilum* (Ap), LIV or TBEV at 168 hpi for selected genes associated with innate immunity and anti-pathogen response. Statistical significance denoted by *P*- or *q*-values highlighted in bold (< 0.05); genes are considered differentially expressed when both *P* and *q* < 0.05

Gene	VectorBase ID	Pathogen	Log_2_ fold-change (168 hpi)	Statistical analysis (*P*-value/*q*-value)
Toll	ISCW022740	Ap	0.34	0.261/0.587
		LIV	2.08	**0.00005/0.001**
		TBEV	1.64	**0.00005/0.001**
Toll	ISCW007727	Ap	-1.96	0.085/0.358
		LIV	-3.85	**0.0078**/0.051
		TBEV	-2.67	**0.0017/0.022**
Toll	ISCW007724	Ap	-0.12	0.729/0.888
		LIV	-1.26	**0.0008/0.009**
		TBEV	-0.77	**0.018**/0.116
Toll	ISCW017724	Ap	0.23	0.607/0.827
		LIV	0.21	0.690/0.815
		TBEV	-0.08	0.886/0.938
MyD88	ISCW008802	Ap	0.44	0.323/0.632
		LIV	-0.36	No test
		TBEV	-0.02	No test
JAK receptor	ISCW016699	Ap	0.85	**0.035**/0.232
		LIV	0.14	No test
		TBEV	0.30	No test
STAT 3	ISCW005692	Ap	0.23	0.451/0.731
		LIV	0.43	0.141/0.328
		TBEV	0.09	0.770/0.874
SOCS	ISCW019435	Ap	0.38	0.356/0.654
		LIV	1.10	**0.009**/0.055
		TBEV	1.21	**0.005/0.046**
Beta transducing Trp-Asp domain-containing protein	ISCW014204	Ap	-0.26	0.507/0.768
		LIV	1.21	**0.003/0.024**
		TBEV	0.39	No test
MAPK	ISCW018301	Ap	0.21	0.557/0.795
		LIV	1.39	**0.0006/0.007**
		TBEV	0.28	0.486/0.680
MAPK-activated protein kinase	ISCW015270	Ap	1.93	**0.00005/0.003**
		LIV	2.16	**0.00005/0.001**
		TBEV	2.10	**0.00005/0.001**
MAPK phosphatase 5	ISCW021412	Ap	1.22	**0.004**/0.077
		LIV	2.29	**0.00005/0.001**
		TBEV	1.35	**0.002/0.026**
FKBP	ISCW006566	Ap	1.10	**0.0002/0.007**
		LIV	1.20	**0.0001/0.002**
		TBEV	0.62	**0.032**/0.163
Slh1	ISCW018611	Ap	-0.13	0.669/0.862
		LIV	0.60	**0.002/0.018**
		TBEV	0.24	0.270/0.503
XBP1	ISCW007440	Ap	1.39	**0.00005/0.003**
		LIV	0.67	**0.0007/0.008**
		TBEV	0.53	**0.014**/0.097

**Fig. 4:**
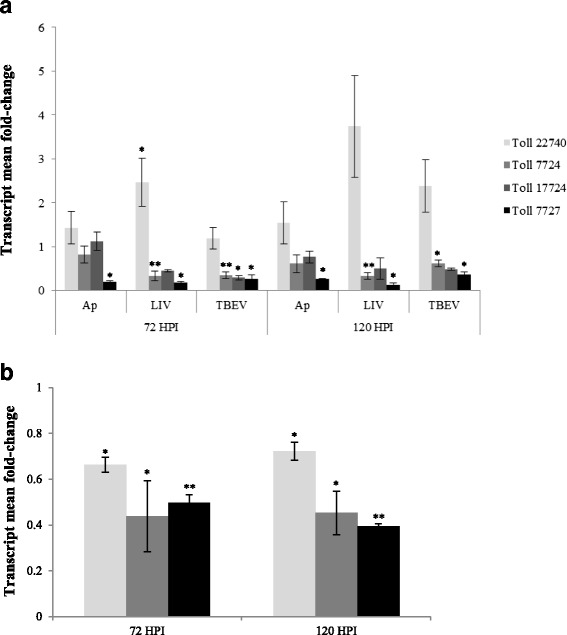
Pathogen-specific effect on biological processes in IRE/CTVM20 cells following infection with tick-borne pathogens at 72 and 120 hpi, as demonstrated by differential expression of (**a**) *toll* genes ISCW022740, ISCW007724, ISCW017724 and ISCW007727, and (**b**) *MyD88* gene ISCW008802, where *A. phagocytophilum*, LIV and TBEV are represented by light *grey* bars, dark grey bars and black *bars* respectively. Statistical significance denoted by *P < 0.05; **P < 0.01

Only flavivirus infection led to significant upregulation of gene expression for toll gene ISCW022740 at 168 hpi (Fig. 3, Table 2). Toll genes ISCW007727 and ISCW7724 were significantly downregulated at 168 hpi following TBEV and LIV infection respectively (Fig. 3, Table 2). There was no change in gene expression for the downstream *MyD88* gene at 168 hpi, although LIV infection appeared to upregulate the gene encoding an antimicrobial peptide (ISCW014204, Beta transducin Trp-Asp domain containing protein) (Fig. 3, Table 2). To further analyse toll gene activation as an initiator of innate immune response, selected genes involved in the Toll pathway were analysed, where RNA from the remaining intermediate time-points at 72 and 120 hpi were assessed using specific primers to determine transcript fold-change (normalised against 16S rRNA). Quantitative RT-PCR for toll gene ISCW022740 demonstrated that this transcript was significantly upregulated at 72 hpi following LIV infection (2.47-fold; SEM = 0.55; *P* = 0.047), with evidence for upregulation at 120 hpi following LIV infection (3.74-fold; SEM = 1.16; *P* = 0.118) and TBEV infection (2.38-fold; SEM = 0.59; *P* = 0.063), when compared to uninfected control cells at 0 hpi (Fig. 4a). In contrast, the remaining toll genes were all significantly downregulated at 72 and 120 hpi following flavivirus infection, in accordance with the RNAseq results at 168 hpi. There was no significant increase in expression of any of the toll genes following *A. phagocytophilum* infection, although toll gene ISCW007727 was significantly downregulated at 72 and 120 hpi (*P* = 0.022 and 0.025, respectively). Despite the upregulation of toll gene expression induced by flavivirus infection, the gene encoding down-stream MyD88, which is activated *via* toll engagement, was significantly downregulated at 72 and 120 hpi following infection with all three pathogens (Fig. 4b, Table 2). Therefore, although there was evidence for antimicrobial peptide gene activation, the selective or limited expression of genes within the Toll pathway suggests that Jak-STAT signalling and activation of the MAP kinase cascade dominate the immune response in this cell line.

In addition to immune genes, a number of genes associated with anti-pathogen responses were differentially expressed (Fig. 3, Table 2). *Anaplasma phagocytophilum* and LIV induced differential expression of genes encoding FK506 binding protein (FKBP) and X-box binding protein 1 (XBP1) (Fig. 3, Table 2); both of these genes are important for immune gene expression, and were significantly upregulated. Additionally, the gene for antiviral helicase Slh1 was differentially expressed following LIV infection only, with significant upregulation suggestive of a direct antiviral response.

## Discussion

The *I. scapularis* genome is the only tick genome fully sequenced and annotated [33, 34], and constitutes a valuable tool for the investigation of interactions between tick-borne pathogens and *Ixodes* spp. ticks [35]. Transcriptional analysis of the *I. scapularis* genome has suggested the involvement of a number of host pathways involved in anti-pathogen responses, including Toll, Jak-STAT and the immune deficiency (Imd) pathway [36–38]. A systems biology approach has previously been utilised to study *A. phagocytophilum* infection in *I. scapularis* ticks, where infection was shown to inhibit apoptosis and upregulate the Jak-STAT pathway, promoting survival and enabling infection to become established [14, 38]. Although the response in tick cells to *A. phagocytophilum* has been well characterised, the response to tick-borne flaviviruses is not yet fully understood. The majority of genes differentially expressed within *I. ricinus* cells infected with LIV and TBEV were associated with metabolic processes (data not shown), an observation shared with previous proteomics studies analysing the infection of *I. scapularis* cells with the related tick-borne flavivirus Langat virus (LGTV) [19]. Similarly, TBEV infection in *I. ricinus* and *I. scapularis* cells has been shown to induce changes in expression of genes associated with a number of processes, including metabolism and immunity [18]. In *Ixodes* spp. tick cells infected with *A. phagocytophilum*, the upregulation of genes associated with apoptosis and cellular stress, including cytochrome *c*, has been previously reported [15], and flavivirus infection appears to activate some of the same genes that could be associated with apoptosis. However, the lack of *Caspase* gene activation suggests selective gene activation, which correlates with microscopic observations of tick cells during the 7-day course of infection with LIV, TBEV (this study), or *A. phagocytophilum* [15], where cell morphology remained unaltered. As vectors of these pathogens, *I. ricinus* ticks therefore have mechanisms for restricting the cellular damage caused by infection, including apoptosis. Hsp70 has previously been shown to inhibit apoptosis through a number of pathways [39–41], suggesting that upregulation of the *hsp70* gene may be involved in the inhibition of apoptosis as a mechanism for host survival. Indeed, knockdown of *hsp70* family genes has been shown to increase LGTV replication in another *I. ricinus* cell line, suggesting a potential antiviral role for these proteins [18]. All three pathogens induced a significant increase in expression of the *hsp70* gene by 168 hpi, and the upregulation of Hsp70 at the protein level has previously been observed in *A. phagocytophilum*-infected tick cells [42]. However, although these data may suggest inhibition of apoptosis, it is possible that the differential expression of these genes could be associated with other cellular responses, such as Hsp70 involvement in protein folding and cellular stress [43].

Innate immune activation is essential for survival following infection, and the infection of tick cells with *A. phagocytophilum* has previously been shown to upregulate the Jak-STAT pathway, with a role in inhibition of apoptosis [14, 15], or the restriction of infection through regulation of antimicrobial peptide expression [37]. Although we could find no evidence of increased expression of Jak or STAT genes following flavivirus infection (Fig. 3), possibly due to limitations in sequence coverage, the differential expression of other genes within the pathway, including *Socs* genes, suggests that selective gene regulation following infection occurs. SOCS inhibit the Jak-STAT pathway, leading to reduced transcription of Jak-STAT genes, and the upregulation of *Socs* gene transcripts has previously been demonstrated in mouse brain following flavivirus infection [44]. Additionally, all three pathogens also induced significant increases in expression of genes within the MAPK cascade. Differential expression of genes involved in both the Jak-STAT and MAPK signalling pathways has also been observed in an *Aedes albopictus* mosquito cell line, following infection with another arthropod-borne virus, bluetongue virus (BTV) [45].

Innate immunity in ticks and tick cells has still not been fully elucidated, but evidence suggests that the Toll signalling pathway is also present in ticks [36]. The lack of detection of toll gene activation following *A. phagocytophilum* infection is unsurprising, since gram-negative bacteria are more likely to initiate the alternative Imd pathway [46]. However, the upregulation of toll gene ISCW022740 following flavivirus infection suggests that the toll pathway is activated during flavivirus infection in tick cells and ticks. This constitutes a host survival mechanism, since the toll pathway has been shown to be important for restricting infection with dengue virus (DENV) in *Aedes aegypti* mosquito cells [47]. However, the downregulation of other toll genes assessed suggests some degree of toll receptor specificity following flavivirus infection, and the downregulation of *MyD88* gene expression following infection with all three pathogens suggests limitations to, or potential inhibition of, toll effector functions. Although not beneficial for host survival, this may enhance infection and benefit tick transmission of pathogens, since MyD88 has been shown to restrict the flaviviruses West Nile virus (WNV) and Japanese encephalitis virus (JEV), in mammalian cells [48, 49]. Furthermore, an absence of MyD88 in mice has been shown to enhance the infectivity of a strain of the spirochaete *Borrelia burgdorferi* when transmitted to *I. scapularis* ticks during feeding [50], suggesting that a lack of expression of this gene enabled an enhancement of infection within tick cells.

Immune activation leads to down-stream upregulation of a number of genes which may be considered to have anti-pathogen functionality. These include the *xbp1* gene, which was upregulated following infection with all three pathogens (although only differentially expressed following *A. phagocytophilum* or LIV infection), consistent with the activation of an innate immune response. The transcription factor XBP1 has multiple functions, including a role in the production of pro-inflammatory cytokines in macrophages, and has been shown to be induced *via* Toll-like receptor (TLR) activation [51]. It has also been shown to contribute to host protection against immune activation in bacterially-infected *Caenorhabditis elegans* nematodes [52]. A similar trend was demonstrated for *fkbp* gene expression, which was also upregulated following infection with all three pathogens. FKBP51, a protein from the FKBP family of proteins, has been shown to interact with TNF receptor associated factor (TRAF) proteins in mammalian cells to facilitate the expression of type I interferon following viral infection [53]. Infection with Sindbis virus (SINV), an alphavirus, has also been shown to alter gene expression for FKBP1 in the midgut of *Ae. aegypti* mosquitoes [54], whilst FKBP1 paralogs have been shown to suppress infection with DENV and WNV in human HeLa cells [55]. FKBPs can therefore be considered to constitute host resistance factors with antiviral potential [56]. Additionally, the antiviral helicase *Slh1* gene was significantly upregulated following LIV infection. Slh1 has been associated with antiviral defence in yeast (*Saccharomyces cerevisiae*), where it has been shown to inhibit translation of viral mRNAs [57]. This provides some evidence that a mechanism may exist within tick cells for limiting flavivirus replication independently of apoptosis, and in addition to immune gene activation.

Host cells respond by increasing apoptosis and activating immune genes for survival. Indeed, *A. phagocytophilum* infection of ISE6 tick cells has been shown to induce other pathways that increase host cell survival, whilst enabling pathogen transmission through limitations to the tick cell response *via* protein mis-folding, suggestive of co-evolution between pathogen and host [42, 58]. There is evidence that selective gene expression in response to bacteria or viruses occurs within tick cells, implying that different cellular recognition proteins are allied to alternative pathways for transcript activation. Viral-induced dsRNA is also known to be recognised in mosquito cells by Dicer-2, which is similar in functionality to mammalian retinoic acid-inducible gene-I (RIG-I), and also activates the Jak-STAT pathway [59]. There may also be activation of alternative immune pathways including RNAi responses in tick cells [17]. However, the response observed in tick cells may vary compared to the response in ticks, although previous studies in tick cell lines have demonstrated that the transcriptional response to *A. phagocytophilum* infection in *I. scapularis* ISE6 cells was similar to that observed in tick haemocytes, whilst the response in *I. ricinus* IRE/CTVM20 cells was more similar to that observed in the tick midgut [15]. Additionally, although these experiments were undertaken at a single temperature of 28 °C, previous studies have shown that variation in temperature can affect tick physiology and tick-pathogen interactions [60]. Therefore, variation in temperature may influence gene expression in tick cell lines, and may lead to alternative gene expression profiles.

## Conclusions

The host response to flavivirus infection in tick cells is complex, and involves the interaction of a number of host mechanisms in order to promote cellular survival, whilst ensuring that virus replication can occur. Infection of *I. ricinus* cells with the tick-borne flaviviruses LIV and TBEV induced an increase in the expression of genes associated with apoptosis, along with the activation of immune genes, including toll, for survival. However, infection with these pathogens was also associated with an increase in gene expression for proteins associated with inhibition of apoptosis, which is promoted by pathogens to increase infection. Therefore, multiple pathways exist within tick cells to provide efficient mechanisms for pathogen control and host survival, and the identification of key genes may contribute to the identification of potential targets for future antiviral strategies. However, differential gene expression in virus-infected cells does not necessarily lead to significant changes in levels of the related proteins, therefore although not in scope for this study, future investigation will include proteomics analysis in order to enhance and validate these transcriptional studies.

## Additional files

Additional file 1: Table S1. Primers used in this study for detection of viral RNA (LIV/TBEV) from infected *I. ricinus* IRE/CTVM20 cells, along with host gene transcripts in RNA extracted from *I. ricinus* IRE/CTVM20 cells infected with *A. phagocytophilum*, LIV or TBEV. (DOC 38 kb)
Additional file 2: Table S2. Build statistics: assessment of sequence depth and quality of alignment for samples taken from *I. ricinus* IRE/CTVM20 cells, uninfected at 0 hpi (samples 1–4), or infected with *A. phagocytophilum* (samples 15–16), LIV (samples 17–18) or TBEV (samples 19–20) at 168 hpi. (DOC 36 kb)

## Abbreviations

Ap: *Anaplasma phagocytophilum*
BTV: Bluetongue virus
cDNA: Complementary DNA
DENV: Dengue virus
DTT: Dithiothreitol
FKBP: FK506 binding protein
HGA: Human granulocytic anaplasmosis
hpi: Hours post-infection
HSP70: Heat shock protein 70
Imd: Immune deficiency
Jak-STAT: Janus kinase-signal transducer and activator of transcription
JEV: Japanese encephalitis virus
LGTV: Langat virus
LIV: Louping ill virus
MAPK: Mitogen-activated protein kinase
M-MLV RT: Maloney murine leukemia virus reverse transcriptase
MOI: Multiplicity of infection
MSP4: Major surface protein 4
MyD88: Myeloid differentiation factor 88
NGS: next generation sequencing
PFU: Plaque forming units
RIG-I: retinoic acid-inducible gene-I
rRNA: Ribosomal RNA
SEM: Standard error of the mean
SINV: Sindbis virus
Slh1: Antiviral helicase
SOCS: Suppressor of cytokine signalling
TBE: Tick-borne encephalitis
TBEV: Tick-borne encephalitis virus
TLR: Toll-like receptor
TPB: Tryptose phosphate broth
TRAF: TNF receptor associated factor
UK: United Kingdom
WNV: West nile virus
XBP1: X-box binding protein 1
