# Supplementary material for: Tick-borne pathogens induce differential expression of genes promoting cell survival and host resistance in Ixodes ricinus cells
Source: Parasit Vectors. 2017 Feb 15;10:81. doi: 10.1186/s13071-017-2011-1 (PMC5312269; doi:10.1186/s13071-017-2011-1)
Supplement: Additional file 1: Table S1. — Primers used in this study for detection of viral RNA (LIV/TBEV) from infected I. ricinus IRE/CTVM20 cells, along with host gene transcripts in RNA extracted from I. ricinus IRE/CTVM20 cells infected with A. phagocytophilum, LIV or TBEV. (DOC 38 kb) [file 13071_2017_2011_MOESM1_ESM.doc]

**Additional file 1: Table S1** Primers used in this study for detection of viral RNA (LIV/TBEV) from infected *I. ricinus* IRE/CTVM20 cells, along with host gene transcripts in RNA extracted from *I. ricinus* IRE/CTVM20 cells infected with *A. phagocytophilum*, LIV or TBEV

| **Target** | **Primer name** | **Primer sequence (5’-3’)** | **Product size (bp)** |
| --- | --- | --- | --- |
| LIV/TBEV | TBEV-E F4 | TGGARCTGGGTGGATGYGT | 229 |
|  | TBEV-E R1 | CAGCCTCGATCACTCTG |  |
| 16S rRNA | Acari for | GGCTCATTAAATCAGTTATTGTCC | 82 |
|  | Acari rev | TGCATGTATTAGCTCTAGAATTGC |  |
| Toll  (ISCW022740) | IxR-Toll22740 for | ATGTCCACGCTGCAAGAG | 171 |
|  | IxR-Toll22740 rev | CATCAGGTTCAGGCCGTA |  |
| Toll  (ISCW007727) | IxR-Toll7727 for | CTCAAGTACAAGCGAGAGAT | 237 |
|  | IxR-Toll7727 rev | GATATCTTGAACTAGATAGCCAC |  |
| Toll  (ISCW007724) | IxR-Toll7724 for | GATCTTCGACGTCTTCCTCTC | 159 |
|  | IxR-Toll7724 rev | GACACCGCTTCATGGATG |  |
| Toll  (ISCW00017724) | IxR-Toll17724 for | AGTGCATTAAGGAAGACGA | 166 |
|  | IxR-Toll17724 rev | CTCTTGGATGATGTCCTG |  |
| MyD88 | IxR-MyD88 for | ACGCATACGTGTGCTACA | 164 |
|  | IxR-MyD88 rev | TATGATGATGGTCTTCTTGC |  |
|  |  |  |  |
|  |  |  |  |
