# Supplementary material for: Tick-borne pathogens induce differential expression of genes promoting cell survival and host resistance in Ixodes ricinus cells
Source: Parasit Vectors. 2017 Feb 15;10:81. doi: 10.1186/s13071-017-2011-1 (PMC5312269; doi:10.1186/s13071-017-2011-1)
Supplement: Additional file 2: Table S2. — Build statistics: assessment of sequence depth and quality of alignment for samples taken from I. ricinus IRE/CTVM20 cells, uninfected at 0 hpi (samples 1–4), or infected with A. phagocytophilum (samples 15–16), LIV (samples 17–18) or TBEV (samples 19–20) at 168 hpi. (DOC 36 kb) [file 13071_2017_2011_MOESM2_ESM.doc]

**Additional file 2: Table S2** Build statistics: assessment of sequence depth and quality of alignment for samples taken from *I. ricinus* IRE/CTVM20 cells, uninfected at 0 hpi (samples 1-4), or infected with *A. phagocytophilum* (samples 15-16), LIV (samples 17-18) or TBEV (samples 19-20) at 168 hpi.

| **Sample ID** | **Mapped Reads (Left/Right)** | **Pairs aligned without duplicates** | **Aligned to genes with no overlap (Exonic)** | **Aligned to genes with overlap into intragenic/intronic region** |
| --- | --- | --- | --- | --- |
| 1 | 7,769,967/6,468,423 | 2871613 | 207,607 (43.31%) | 101,709 (21.22%) |
| 2 | 8,084,370/7,055,360 | 3421027 | 214,440 (38.34%) | 142,895 (25.55%) |
| 3 | 6,620,401/5,356,230 | 2552849 | 193,292 (46.29%) | 76,547 (18.33%) |
| 4 | 5,395,954/4,938,827 | 2007933 | 182,982 (42.74%) | 84,272 (19.68%) |
| 15 | 7,233,127/6,172,478 | 2652424 | 218,575 (39.87%) | 151,461 (27.65%) |
| 16 | 8,202,079/7,135,160 | 3491641 | 245,046 (40.08%) | 147,488 (24.12%) |
| 17 | 8,509,598/7,565,552 | 3450457 | 259,756 (48.54%) | 95,169 (17.78%) |
| 18 | 7,432,541/6,260,493 | 2369215 | 232,828 (50.65%) | 75,116 (16.34%) |
| 19 | 7,477,049/5,862,591 | 2323838 | 245,851 (43.05%) | 149,073 (26.1%) |
| 20 | 10,043,015/8,867,589 | 4772418 | 252,437 (42.21%) | 139,310 (23.39%) |
